# Inhibition of uncoupling protein 2 with genipin exacerbates palmitate-induced hepatic steatosis

**DOI:** 10.1186/1476-511X-11-154

**Published:** 2012-11-14

**Authors:** Shuangtao Ma, Dachun Yang, De Li, Yan Tan, Bing Tang, Yongjian Yang

**Affiliations:** 1 Department of Cardiology, General Hospital of PLA Chengdu Military Area Command, 270 Rongdu Rd., Tianhui Town, Jinniu District, Chengdu, 610083, Sichuan Province, People’s Republic of China

## Abstract

**Background:**

Uncoupling protein 2 (UCP2) was reported to be involved in lipid metabolism through regulating the production of superoxide anion. However, the role of UCP2 in hepatocytes steatosis has not been determined. We hypothesized that UCP2 might regulate hepatic steatosis via suppressing oxidative stress.

**Results:**

We tested this hypothesis in an *in vitro* model of hepatocytic steatosis in HepG2 cell lines induced by palmitic acid (PA). We found that treatment with PA induced an obvious lipid accumulation in HepG2 cells and a significant increase in intracellular triglyceride content. Moreover, the specific inhibition of UCP2 by genipin remarkably exacerbated PA-induced hepatocytes steatosis. Interestingly, the PA-induced superoxide overproduction can also be enhanced by incubation with genipin. In addition, administration with the antioxidant tempol abolished genipin-induced increase in intracellular lipid deposition. We further found that genipin significantly increased the protein expression of fatty acid translocase (FAT)/CD36.

**Conclusions:**

These findings suggest that UCP2 plays a protective role in PA-induced hepatocytic steatosis through ameliorating oxidative stress.

## Background

Hepatic steatosis is an important process in the development of nonalcoholic fatty liver disease that is a major component of metabolic syndrome and a significant risk factor of cardiovascular disease [1]. The accumulation of excess neutral fat within hepatocytes is closely related to obesity and overweight [2], suggesting hepatic steatosis mostly results from metabolic disturbance of lipids [3]. It was reported that increased level of free fatty acid (FA) is associated with the onset of fatty liver disease and hepatic steatosis [4]. However, the molecular mechanism underlying FA-induced hepatic fat deposition remains to be determined.

Uncoupling protein 2 (UCP2) belongs to the mitochondrial carrier family and has been thought to be a metabolic sensor coupling excess FA to lipotoxity [5]. Not only the plasma FA levels are accompanied by the increased UCP2 expression but also the FA can stimulate the activity of the promoter regions of UCP2 gene [6]. Moreover, the UCP2 is suggested to promote mitochondrial FA oxidation while limiting mitochondrial catabolism of pyruvate [7]. These findings indicate that UCP2 might be involved in the development of FA-induced hepatic steatosis.

Attenuation of hepatic oxidative stress inhibits fat deposition, indicating that reactive oxygen species (ROS) might play a central role in the development of hepatic steatosis [8]. UCP2 has been linked to mitochondria-derived ROS production. The previous studies have demonstrated that UCP2 ablation can enhance the oxidative stress though decreasing the proton leak [9]. In addition, ROS is generated during the metabolism of free FA in mitochondria [10]. Thus, UCP2 might be involved in hepatic steatosis via regulating ROS production in the process of excess FA oxidation. As UCP2 interacts to the FA translocase (FAT)/CD36, the uptake of FA might also be regulated by UCP2 [11].

Therefore, we hypothesized that UCP2 might regulate hepatic steatosis though a ROS-dependent pathway. In the present study, we present a cellular model of hepatic steatosis induced by palmitic acid (PA). We report that inhibition of UCP2 by genipin increases ROS production and enhances hepatocytic lipid deposition induced by PA. We also found that genipin-mediated lipid deposition is linked to the upregulation of FAT/CD36.

## Results

### Inhibition of UCP2 enhances PA-induced hepatic steatosis

**Figure 1:**
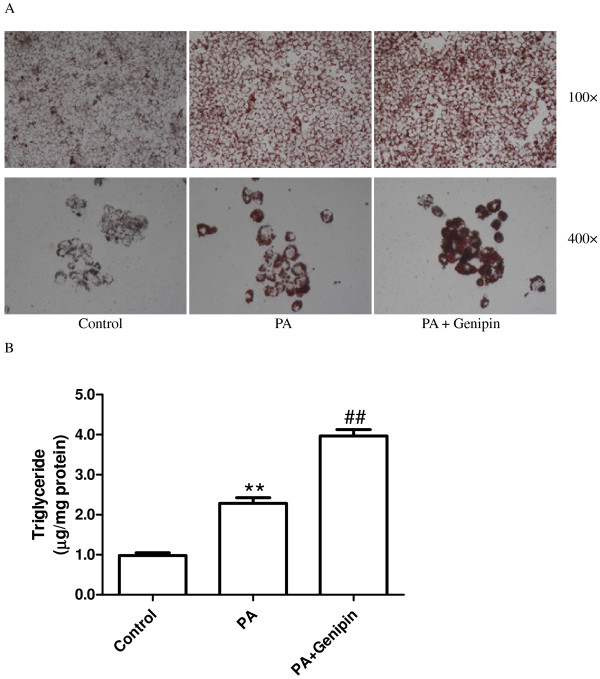
**Genipin enhances palmitic acid-induced hepatic steatosis.** (**A**) Neutral fat accumulations after 24 h of exposure to control medium, palmitic acid (PA, 250 μmol/L) and PA plus genipin (5 μmol/L) are shown by staining of HepG2 cells with Oil-Red O. (**B**) Triglyceride level normalized to cellular protein content in HepG2 was determined. Data are expressed as mean ± SEM. ** *p* < 0.01 compared with the control group, ^##^*p* < 0.01 compared with the cells treated with PA. PA, palmitic acid.

Intracellular lipid accumulation was examined using Oil Red O staining. Treating cultured hepatocytes with PA (250 μmol/L) medium for 24 h resulted in steatosis; in contrast, little lipid droplet was found in the untreated cells (Figure 1A). When cultured hepatocytes were incubated with PA plus UCP2 inhibitor genipin (5 μmol/L), the cellular lipid inclusions were significantly increased compared with cells treated with PA alone (Figure 1A). Intracellular triglyceride level was significantly increased from control by treatment for 24 h with PA (*p* < 0.01, Figure 1B) and was further elevated by incubating with genipin (*p* < 0.01, Figure 1B).

### Inhibition of UCP2 enhances PA-induced oxidative stress

**Figure 2:**
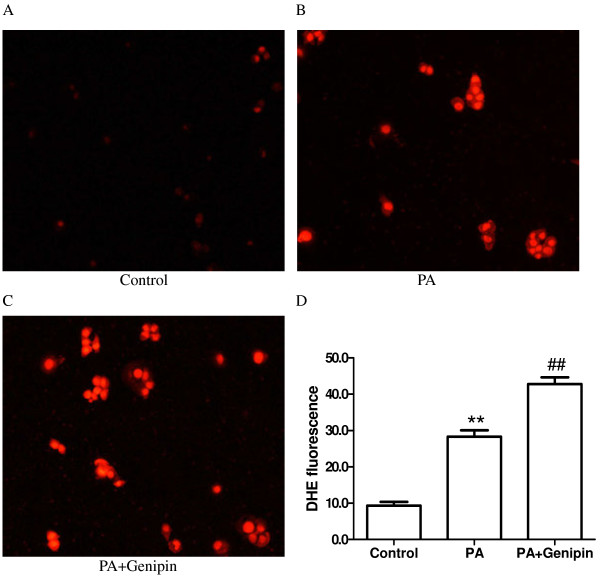
**Genipin enhances palmitic acid-induced superoxide production.** Intracellular superoxide anion was detected by DHE staining. HepG2 cells were exposed to control medium (**A**), palmitic acid (PA, 250 μmol/L) (**B**) and PA plus genipin (5 μmol/L) (**C**) for 24 h. (**D**) Summarized data showing the average fluorescence intensity in cells from each group. Data are means ± SEM from six individual experiments. ** *p* < 0.01 compared with the control group, ^##^*p* < 0.01 compared with the cells treated with PA. PA, palmitic acid; DHE, dihydroethidium.

Superoxide anion production in hepatocyte was assessed by dihydroethidium (DHE) staining. We found that DHE fluorescence was significantly higher in PA-treated cells than untreated ones (Figure 2A, B and D). Additionally, DHE fluorescence in hepatocyte treated with PA plus genipin was markedly higher than PA-treated cells (Figure 2B, C and D).

### Antioxidant abolishes genipin-induced hepatic steatosis

**Figure 3:**
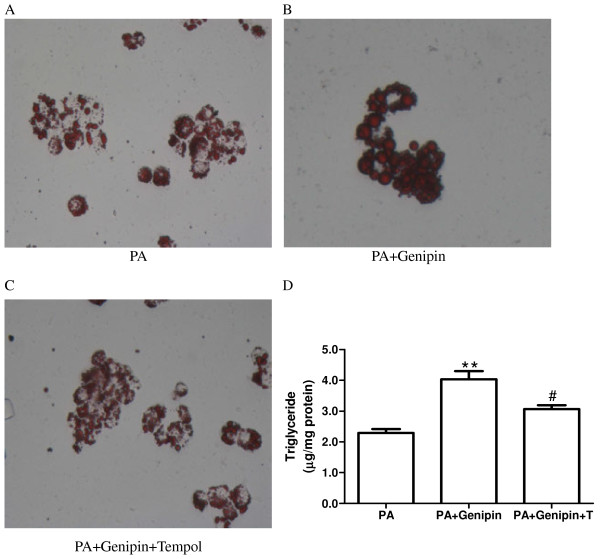
**Tempol blocks genipin-mediated hepatic steatosis.** Neutral fat accumulations after 24 h of exposure to palmitic acid (PA, 250 μmol/L) (**A**), PA plus genipin (5 μmol/L) (**B**) and PA plus genipin and tempol (1 mmol/L) (**C**) are shown by staining of HepG2 cells with Oil-Red O. (**D**) Triglyceride level normalized to cellular protein content in HepG2 was determined. Data are expressed as mean ± SEM. ** *p* < 0.01 compared with the PA group, ^#^*p* < 0.05 compared with the cells treated with PA plus genipin. PA, palmitic acid; T, tempol.

To confirm the central role of oxidative stress in UCP2 inhibition-mediated enhancement of lipid deposition in hepatocytes, the antioxidant tempol was applied. We found that blocking oxidative stress with tempol reversed genipin-induced further increase in intracellular lipid deposition (*p* < 0.05, Figure 3A, B, C and D).

### Inhibition of UCP2 upregurates FAT/CD36

**Figure 4:**
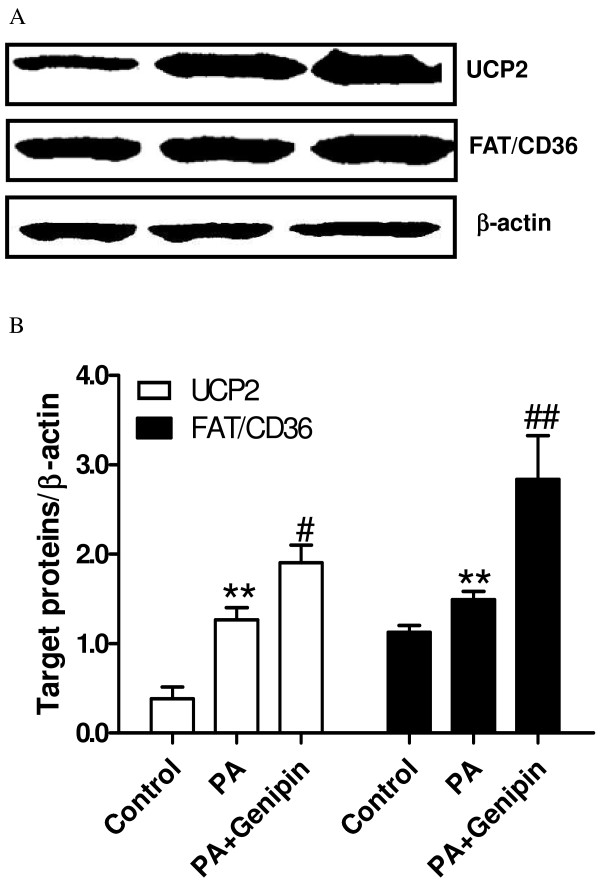
**Genipin upregulates UCP2 and FAT/CD36.** (**A**) Western blot analysis of UCP2 and FAT/CD36 expressions. The protein was isolated from the HepG2 cells after 24 h of exposure to control medium, palmitic acid (PA, 250 μmol/L) and PA plus genipin (5 μmol/L). Equal protein loading was confirmed using β-actin antibody. (**B**) Target proteins/β-actin is shown in the bar graph. ** *p* < 0.01 vs. control group, ^#^*p* < 0.05, ^##^*p* < 0.01 vs. PA group (n = 4).

Treatment with PA significantly increased the protein expressions of UCP2 and FAT/CD36 in cultured hepatocytes (*p* < 0.01, Figure 4A and B). Surprisingly, inhibition of UCP2-mediated proton leak with genipin further increased the expression levels of UCP2 and FAT/CD36 (*p* < 0.05 or *p* < 0.01, Figure 4A and B).

## Discussion

In this study, we assessed the role of UCP2 in regulating ROS production in cultured hepatocytes under the condition of FA treatment and established a critical role of UCP2 in the pathogenesis of hepatic steatosis. We showed that nutrient excess in the form of high PA medium resulted in a rapid development of hepatic steatosis that was enhanced by inhibiting UCP2 with genipin. We also demonstrated that PA caused production of superoxide was enhanced by genipin, while antioxidant tempol blocked genipin-mediated steatosis. In addition, we found that protein expressions of UCP2 and FAT/CD36 were significantly upregulated by both PA and genipin.

The UCP2 protein expression has been linked to the occurrence of nonalcoholic fatty liver disease [12]. The expression level of UCP2 in normal hepatocyte is very low but can be significantly upregulated under the obese condition [13]. Several studies have demonstrated that hepatic steatosis is associated with increased expression of UCP2 expression [14]. However, the exact role of UCP2 in the development of hepatic lipid deposition has not been determined. The present study shows that inhibition of UCP2 accelerates hepatic steatosis, indicating that UCP2 might play a compensatory protective role in PA-induced hepatic fat deposition. This finding suggests that the drugs upregulating UCP2 might become a novel strategy for fighting nonalcoholic fatty liver disease.

The current concept suggests that the oxidative stress plays an important role in the development of hepatic steatosis. UCP2, a well-established uncoupling protein, has been viewed as an inhibitor of mitochondrial ROS production [15]. The present study demonstrates that the tempol, a well-known antioxidant, blocks UCP2 inhibition mediated hepatic steatosis. This finding suggests that UCP2 inhibition-caused liver fat deposition is dependent on the overproduction of superoxide.

UCP2 has been implicated in physiological and pathological processes related to lipid metabolism, especially in the uptake and oxidation of free FA [16,17]. The enzymes responsible for FA metabolism, such as peroxisome proliferator-activated receptor α and FAT/CD36, were linked to UCP2 [18]. The present study found that UCP2 inhibition-associated lipid deposition is related to the upregulation of FAT/CD36, indicating the uptake of PA by hepatocyte is increased. The genipin-induced upregulation of UCP2 might be a compensatory effect.

## Conclusions

In conclusion, the present results demonstrate that UCP2 plays a protective role in free FA-induced hepatic steatosis. Moreover, the UCP2-mediated lipid deposition is attributed to the production of superoxide. The present findings may contribute to the insights into the clinical prevention and management of nonalcoholic fatty liver disease.

## Methods

### Cell culture and treatment

HepG2 cells were obtained from the Shanghai Institute of Cell Biology (Shanghai, China). Cells were grown in Dulbecco’s modified eagle’s medium (DMEM; Invitrogen Co., Carlsbad, California, USA) with 10% FBS in the presence of 100 U/mL penicillin and 100 μg/mL streptomycin and maintained at 37°C in a humidified atmosphere containing 5% (v/v) CO_2_.

### Steatosis analysis

Cultured HepG2 cell were plated on cover slides in six-well plates and maintained in culture media until the cells reached 95% confluence. Experiment 1: The cells were incubated for 24 h with control medium, PA medium (addition of 250 μmol/L PA) and PA plus genipin (addition of 5 μmol/L genipin) supplemented with identical glucose and serum concentrations. This PA concentration was used because it has been successfully used in the previous studies. After that, the cells were washed with phosphate-buffered saline, fixed with 4% paraformaldehyde, and stained with Oil Red O, which detects neutral lipids such as cholesteryl esters and triglyceride [19]. Intracellular triglyceride was detected. Experiment 2: The cells were divided into three groups: PA (treated with 250 μmol/L of PA), PA + genipin (treated with 250 μmol/L of OA plus 5 μmol/L of genipin) and PA + genipin + tempol (treated with 250 μmol/L of OA, 5 μmol/L of genipin and 1 mmol/L of tempol). After incubation for 24 h, Oil Red O staining of the cells was performed, and the intracellular triglyceride was measured. The cells were collected for the detection of UCP2 and FAT/CD36 expressions.

### Dihydroethidium assay

To assess superoxide production, the HepG2 cells were cultured and treated as above. The medium was removed, and the cells were rinsed twice with PBS. After that, the cells were incubated in the dark with dihydroethidium (DHE; Sigma-Aldrich, St Louis, MO) diluted in Krebs (40 μmol/l) for 30 min at 37°C followed by a 15 min wash in DHE-free Krebs. To quantitate the DHE fluorescence, the glass slides were placed under the Leica DM LB2 Fluorescent Microscope (Leica, Wetzlar, Germany) outfitted with a rhodamine filter set [10].

### Western blotting

Cell lysate was prepared by lysing the cells with buffer containing 1% Triton X-100, 150 mM NaCl, 1 mM EDTA, 2.5 mM sodium pyrophosphate, 1 mM β-glycerophosphate, 1 mM Na3VO4, 1 μg/mL leupeptin, 1 μg/mL aprotinin, and 20 mM Tris (pH 7.5). Protein concentration was determined by Bio-Rad protein assay reagent (Bio-Rad Laboratories, USA). Lysate was electrophoretically transferred onto a nitrocellulose membrane and immunoblotted with rabbit anti-UCP2 and FAT/CD36 IgG (1:500 dilution, Sigma-Aldrich Co., USA). The blots were incubated with a horseradish peroxidase-conjugated secondary antibody (1:1000 dilution, Santa Cruz Biotechnology, USA), and the bound antibody was visualized using a colored reaction. The relative band intensity was quantified by the use of Quantity One software (Bio-Rad). Equal loading of protein was confirmed by measuring β-actin expression [20].

### Statistical analysis

Data are presented as mean ± SEM. Comparisons between groups were determined by one-way ANOVA with a post hoc Student’s *t*-test (SPSS Inc., Chicago, IL). Probabilities of *p* < 0.05 were considered statistically significant.

## Abbreviations

FA: Fatty acid; FAT/CD36: Fatty acid translocase; PA: Palmitic acid; ROS: Reactive oxygen species; UCP2: Uncoupling protein 2; DHE: Dihydroethidium.

## Competing interests

The authors declare that they have no competing interests.

## Authors’ contributions

SM carried out the Oil Red O staining and drafted the manuscript. DY carried out the cell studies. DL participated in the molecular studies. YT participated in the design of the study. BT performed the statistical analysis. YY conceived of the study, and participated in its design and coordination and helped to draft the manuscript. All authors read and approved the final manuscript.
